# Incidental Finding of Combined Factor V and VIII Deficiency in the Setting of a Preoperative Evaluation: A Case Report

**DOI:** 10.7759/cureus.91406

**Published:** 2025-09-01

**Authors:** Joseph L Luzarraga, Jeremy I Purow, Aron Berkman

**Affiliations:** 1 Medicine, Florida International University, Herbert Wertheim College of Medicine, Miami, USA; 2 Public Health, Florida International University, Herbert Wertheim College of Medicine, Miami, USA; 3 Hematology and Oncology, Florida International University, Herbert Wertheim College of Medicine, Miami, USA

**Keywords:** clotting factor v deficiency, clotting factor viii deficiency, factor v and viii deficiency, genetics, lman1, mcfd2, medical education, pre-operative evaluation, coagulopathy

## Abstract

We present a case of a patient who was found to have incidental deficiencies of combined factor V (F5) and factor VIII (F8) identified on pre-operative screening. Our patient is a 61-year-old male with a past medical history of hyperparathyroidism, hypercalcemia, and mild kidney dysfunction. He was referred to the hematology clinic as he was found to have an abnormal activated partial thromboplastin time (aPTT) while being evaluated preoperatively for a parathyroidectomy. The patient did not have any significant history of bleeding, bruising, or hemarthrosis but recalls occasional nosebleeds in childhood. The patient was screened with PT/aPTT by his primary care prior to surgery. This revealed a mildly prolonged aPTT and a normal prothrombin time (PT), and the patient was referred to hematology. Further studies one week later revealed decreased F5 and F8 activity. von Willebrand Factor antigen levels were normal. CBC, protein C and S activity, and antiphospholipid antibody screening were normal as well. This is postulated to arise from a monogenic mechanism, classically through biallelic variants in LMAN1 or MCFD2, leading to combined F5 and F8 deficiency. Rarely, it can be of digenic inheritance with separate defects in F5 and F8. We outline considerations for perioperative evaluation and family counseling in the setting of a rare coagulopathy.

## Introduction

Bleeding disorders broadly classify a large group of diseases characterized by an increased propensity toward episodes of bleeding due to aberrations in the clotting cascade. These disorders can be separated into two classes depending on whether there are aberrations in primary or secondary hemostasis. Examples of diseases of primary clotting are von Willebrand’s disease, Bernard Soulier's disease, and Glanzmann thrombasthenia, which are characterized by platelet dysfunction. Diseases of the secondary clotting cascade include clotting factor deficiency or dysfunction. Clotting factors stabilize the platelet plug by converting fibrinogen to fibrin, creating a mesh. Examples of these diseases include hemophilia A, hemophilia B, vitamin K deficiency, disseminated intravascular coagulation, and coagulation factor inhibitor diseases [1].

Prothrombin time (PT) primarily assesses the extrinsic and common pathways, while activated partial thromboplastin time (aPTT) evaluates the intrinsic and common pathways [2]. F8 functions as a cofactor for FIXa, primarily prolonging aPTT when activity is low [3]. F5 functions as a cofactor for FXa, within the prothrombinase complex, potentially leading to prolongations in both PT and aPTT. Consequently, the degree of reduction of F5 may lead to abnormalities in either metric [4]. 

Isolated F5 deficiency is a rare autosomal recessive bleeding disorder affecting about 1:1,000,000 worldwide, generally as a result of loss-of-function variants in F5, resulting in a primary deficiency [5]. Clinical heterogeneity is common, ranging from asymptomatic deficiency to severe bleeding [5]. F8 deficiency or hemophilia A has an X-linked recessive inheritance and therefore a male prevalence of about 1:5,000-10,000 individuals [6]. This disease is characterized by hemarthrosis and bleeding into the muscles and soft tissues. Loss-of-function variants in F8 lead to primary hemophilia A. Initial assessment includes obtaining F8 assays.

Of all patients with coagulation disorders, only 3% present with combined deficiencies in both F5 and F8 [7]. Multiple papers have identified genetic variants that have been associated with combined F5 and F8 deficiencies, including the LMAN1 and MCFD2 [7,8,9]. LMAN1 (lecithin mannose-binding 1, on chromosome 18q21) and MCFD2 (multiple coagulation factor deficiency 2, on chromosome 2p21) encode for intracellular transport proteins. Described predominantly within consanguineous families and certain founder populations, it is typically inherited in an autosomal recessive manner. They are components of the endoplasmic reticulum (ER)-Golgi intermediate compartment (ERGIC-53) involved in F5 and F8 intracellular transport [10]. MCFD2 interacts with ERGIC53 in a calcium-dependent manner, which may be relevant to our case in the setting of hyperparathyroidism, and the complex recycles between the ER and the ERGIC. The ERGIC-53/MCFD2 complex is believed to capture F5 and F8 in the ER and to package the two coagulation factors into transport vesicles that mediate transport to the ERGIC. These indicate that LMAN1 and MCFD2 must function as a unit in order to transport F5 and F8 [11,12]. It has been posited that these genes play a role in the cellular transport of F5 and F8 from the endoplasmic reticulum to the Golgi apparatus [10]. 

Most patients have mild or moderate bleeding and do not necessarily require treatment with fresh frozen plasma [7,8]. However, some patients require more aggressive management due to the propensity to have more severe bleeding episodes [9]. Female patients often have significant complications due to heavy menstrual bleeding and require careful management and planning to prevent potentially life-threatening bleeding associated with pregnancy [9]. We present a rare case of a patient with a combined F5 and F8 deficiency with mostly mild bleeding symptoms.

## Case presentation

We present a case of a 61-year-old Nicaraguan-born male with a pertinent history of hyperparathyroidism and hypercalcemia who was found to have an abnormal aPTT and unremarkable PT during a preoperative work-up for a parathyroidectomy. He has a relatively uncomplicated medical history besides mildly decreased kidney function (elevated kidney enzymes), a prior history of herpes simplex 1 managed with acyclovir, and a history of gout. At the time of presentation, the patient was taking allopurinol and colchicine; no other medications or supplements were noted. He was a former smoker of 1 pack per day for 15 years but quit 35 years ago. 

Hematologically, he states that he has a history of frequent nosebleeds during childhood but denies any other complications with bleeding or clotting. He underwent surgery to repair a leg fracture in childhood without complication. The patient states that his son was diagnosed with von Willebrand’s disease but notes no other significant family history or bleeding history. 

When the patient was initially referred to hematology, coagulation studies had already been conducted. Screening aPTT was 36 seconds (normal: 25-35 sec) and PT was 11.6 seconds (normal: 11-13.5) by the patient’s primary care physician. This elevation prompted cautious repetition and further analysis of his coagulation studies prior to his initial hematology visit. Six days later, his aPTT 35 and PT 11.4 had normalized. However, the extended study demonstrated that F5 and F8 activity were notably low at 57% and 43%, respectively. Von Willebrand factor (vWF) antigen was 80%, and protein C and S activity were normal (Table 1). The patient tested negative for the antiphospholipid antibody screen (including lupus anticoagulant) (Table 2). The patient’s complete blood count (CBC) was within normal limits with a white blood cell count (WBC) of 6.8 thousand/uL, hematocrit (HCT) of 47%, mean corpuscular volume (MCV) of 89.7 fL, and a platelet count of 179 thousand/uL (Table 3). The differential was normal. His calcium was elevated to 11.2, likely in the setting of mild renal disease and hyperparathyroidism. Liver function tests (LFTs) were mildly elevated with aspartate aminotransferase (AST) 38 and alanine transaminase (ALT) 56 without evidence of infectious hepatitis B or C. 

**Table 1:** Coagulation studies aPTT: activated partial thromboplastin time, PT: prothrombin time

Coagulation studies	Value	Reference range
aPTT	34 seconds	25-35 seconds
PT	11.1 seconds	11-13.5 seconds
Factor II activity	90%	70-120%
Factor V activity	57%	70-140%
Factor VIII activity	46%	50-150%
Factor XI activity	97%	70-150%
Von Willebrand factor antigen	72%	50-150%
Protein C activity protein S activity	92% 83%	70-180% 70-150%
D-dimer	.28 μg/mL	< .5>
Fibrinogen	266 mg/dL	200-400 mg/dL
Mixing study PTT	46 seconds	25-35 seconds
Mixing study PT	12.5 seconds	11-13.5 seconds

**Table 2:** Additional labs HIV: human immunodeficiency virus, CRP: C-reactive protein

Additional labs	Value	Reference range
Antiphospholipid antibody	Negative	Negative
Rheumatoid factor	<14 IU/mL	<14 IU/mL
Lupus anticoagulant	Negative	Negative
HIV	Negative	Negative
Hepatitis B surface antigen	Negative	Negative
Hepatitis C surface antibody	Negative	Negative
CRP	.4 mg/L	<10 mg/L

**Table 3:** CBC results CBC: complete blood count, WBCs: white blood cells, Hgb: hemoglobin, Hct: hematocrit, MCV: mean corpuscular volume

CBC	Value	Reference range
WBCs	7.6 x 103 /μL	(4-11) x 103/μL
Hgb	15.6 g/dL	13.5-17.5 g/dL
Hct	47.4%	41-53%
MCV	94.8 fL	80-100 fL
Platelets	201,000/μL	150,00-400,000/μL

Liver function tests, platelet count, fibrinogen, and vitamin K-dependent factor activities were not consistent with an acquired cause. The patient’s physical exam was unremarkable, and there was no evidence of petechiae, active bleeding, or bruising. 

Given the patient's clinical stability and lack of history of significant bleeding, the patient was granted preoperative clearance. In this case, no intervention was necessary, and the patient was advised to pursue surgery with precautions for intraoperative bleeding on standby. 

## Discussion

**Figure 1:**
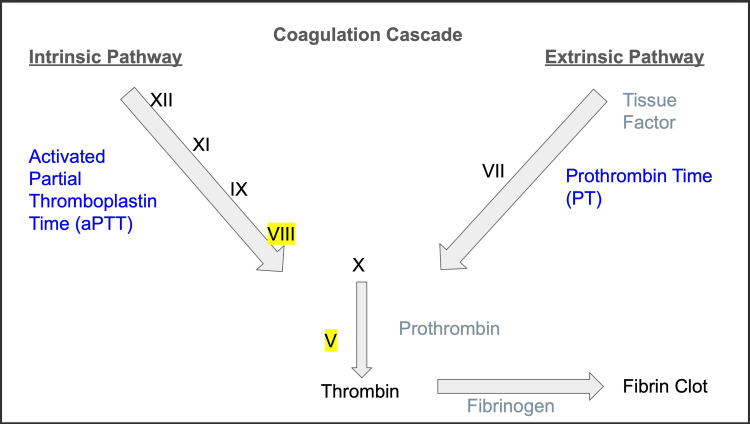
Coagulation cascade

The patient’s workup was largely normal aside from an elevated aPTT that was secondary to decreased F5 and F8 activity. While hepatic dysfunction may lead to factor deficiencies, this is highly improbable in our patient since this does not usually lead to a selective deficiency only in factors F5 and F8. A more plausible etiology for this deficiency is a genetic variant in a factor production pathway, leading to a combined F5 and F8 deficiency. The coagulation cascade is shown in Figure 1.

This is most commonly the result of the monogenic combined F5 and F8 disorder, caused by biallelic variants in LMAN1 or MCFD2. These genes encode a cargo receptor complex instrumental for intracellular ER-to-Golgi transport of both F5 and F8 via COPII-coated vesicles [7,10,11]. In such cases, both factors are reduced by a similar extent, often between 5% and 30%. A more rare possibility is a true digenic inheritance or independent pathogenic variants in F5 and F8.

Isolated F8 deficiency would prolong only aPTT, while significant F5 deficiency would cause aberrations in both PT and aPTT. Mixing studies are essential to evaluate for an inhibitor, such as a lupus anticoagulant or FVIII inhibitor. Correction of these studies indicates the absence of such. In patients with F5 and F8 deficiency, both factors should be reduced to a similar extent, without changes in vWF antigen activity. Here, the genetic disruption of intracellular trafficking rather than vWF stabilization leads to coagulopathy. 

An important piece of the patient’s presentation lies in the family history. The patient notes that his son had been diagnosed with von Willebrand’s disease. A family history of bleeding disorders may suggest an inherited predisposition. Further genetic testing and pedigree analysis should be pursued to localize the specific aberration present in this patient’s presentation and how it may correlate with his son’s history. At this point, there is no evidence that these genes have any relation to von Willebrand’s disease. However, this patient’s family history also calls for additional research into LMAN1 and MCFD2 to further investigate the mechanisms involved in coagulation, which these genes may code for. 

Given that most patients with combined F5 and F8 deficiency do not experience severe bleeding, they often do not need factor replacement therapy for their condition. However, some patients may require factor replacement therapy prior to surgery or other precautionary measures. In our case, hematologic clearance was provided, and elective surgery was scheduled with precautionary measures, such as blood products on standby, in the event of severe bleeding. 

This case is significant as it contributes to the limited literature regarding the rare finding of combined F5 and F8 deficiency [7,8]. This case also contributes data that indicates the clinical heterogeneity of this condition, which may range from those who are asymptomatic or have a mild bleeding phenotype to those who have severe bleeding, such as heavy menstrual bleeding and severe obstetric complications [9].

## Conclusions

This case highlighted a rare and diagnostically challenging presentation, underscoring the importance of thorough preoperative screening of even asymptomatic patients. Findings of combined F5 and F8 deficiency in a clinically asymptomatic adult reflect a rare instance where preoperative evaluation was imperative. As in our patient, even mild alterations in coagulation studies may call for additional screening to identify a root cause and stratify risk of bleeding. However, our patient’s mild prolongation of coagulation studies suggests a subclinical phenotype, highlighting that even minor findings may be important. Careful history taking of unclear etiologies is essential to prevent catastrophic bleeding during high-risk procedures such as cardiovascular and neurosurgical procedures. Lastly, further genetic study is warranted to elucidate any relation between this patient’s presentation and the von Willebrand’s disease of his son. This will help us know if those with mutations in LMAN or MCFD2 or their family members are predisposed to other coagulation disorders.
